# Multifaceted Impact of Host C–C Chemokine CCL2 in the Immuno-Pathogenesis of HIV-1/*M. tuberculosis* Co-Infection

**DOI:** 10.3389/fimmu.2013.00312

**Published:** 2013-10-04

**Authors:** A. Wahid Ansari, Adeeba Kamarulzaman, Reinhold E. Schmidt

**Affiliations:** ^1^Faculty of Medicine, Centre of Excellence for Research in AIDS (CERiA), University of Malaya, Kuala Lumpur, Malaysia; ^2^Clinic for Immunology and Rheumatology, Hannover Medical School, Hannover, Germany

**Keywords:** HIV/Mtb co-infection, immuno-pathogenesis, CCL2, macrophages, granuloma, CD4+ T cells, viremia

## Abstract

Active tuberculosis remains the leading cause of death among the HIV-1 seropositive individuals. Although significant success has been achieved in bringing down the number of HIV/AIDS-related mortality and morbidity following implementation of highly active anti-retroviral therapy (HAART). Yet, co-infection of *Mycobacterium tuberculosis* (Mtb) has posed severe clinical and preventive challenges in our efforts to eradicate the virus from the body. Both HIV-1 and Mtb commonly infect macrophages and trigger production of host inflammatory mediators that subsequently regulate the immune response and disease pathogenesis. These inflammatory mediators can impose beneficial or detrimental effects on each pathogen and eventually on host. Among these, inflammatory C–C chemokines play a central role in HIV-1 and Mtb pathogenesis. However, their role in lung-specific mechanisms of HIV-1 and Mtb interaction are poorly understood. In this review we highlight current view on the role of C–C chemokines, more precisely CCL2, on HIV-1: Mtb interaction, potential mechanisms of action and adverse clinical consequences in a setting HIV-1/Mtb co-infection. Targeting common chemokine regulators of HIV-1/Mtb pathogenesis can be an attractive and potential anti-inflammatory intervention in HIV/AIDS-related comorbidities.

## Introduction

According to United Nations Program on HIV/AIDS (1) nearly 14 million individuals are living with HIV-1/ *Mycobacterium tuberculosis* (Mtb) co-infection (http://www.unaids.org/documents/20101123_GlobalReport_Chap2_em.pdf), estimating around 26% of HIV/AIDS-related deaths each year (2). The host immune response elicited against these pathogens can impose beneficial or detrimental effects on each other and the host. HIV/AIDS is characterized by severe immune dysfunction associated with marked reduction in CD4+ T cell counts and high plasma HIV-1 viral load. Under such immune-deficient condition HIV-1+ individuals become susceptible to infection by opportunistic pathogens, including Mtb. Pathologically, both HIV-1 and Mtb infect alveolar macrophages in a setting of pulmonary co-infection. Seminal contributions have been made in past decades to understand the role of host derived soluble factors in HIV-1 and Mtb mono-infections (3–8), while lesser is known in HIV-1/Mtb co-infection setting. Nonetheless, clinical data has supported the production of some of the common soluble factors induced by these pathogens. For example production of pro-inflammatory mediators like IFN-γ, TNF-α, and CCL2 (MCP-1) by both HIV-1 and Mtb contribute significantly in disease control.

Chemokines are small molecular weight proteins involved in immuno-regulatory and inflammatory functions (9, 10). Based on their N-terminal cysteine residues they are categorized into: C–, C–C, C–X–C, and C–X_3_–C sub-families (10, 11). However, based on function additional classification has also been suggested into homeostatic or inflammatory chemokines (9). For example, homeostatic C–C chemokines such as CCL19 and CCL21 control homing of CCR7+ dendritic cells (DCs) and lymphocytes in the secondary lymphoid organs for optimal immune reactions (11). While inflammatory chemokines, CCL3 (MIP-1α), CCL3 (MIP-1β), CCL5 (RANTES), CXCL8 (IL-8), CXCL9 (MIG), CXCL10 (IP-10), and CXCL11 (I-TAC) participate in inflammation, autoimmune disorders, and malignancies (12–16). The pro-inflammatory chemokine CCL2 is linked to a number of human acute and chronic viral infections including HIV-1 (3, 17, 18). In addition to HIV-1+ individuals, a higher CCL2 levels are also detected in the broncho alveolar lavage (BAL) fluid of pulmonary TB patients (19) and pleural fluid of both HIV-1 infected and un-infected patients (20). Thus induction of CCL2 by both pathogens is an interesting aspect that needs to be addressed in a setting of HIV-1/Mtb co-infection.

The most striking feature of Mtb infection is the formation of granuloma, a highly organized cellular structure composed of macrophages, T cells, NK cells, B-cells, neutrophils, and DCs. Functionally granuloma restricts the Mtb bacilli within this specialized microenvironment. Several hypotheses are drawn to describe the mechanism by which HIV-1 increases the risk of TB reactivation. Some of the potential mechanisms include (1) persistent HIV-1 replication in the lung causes immune dysfunction (20). (2) HIV-1 induced CD4+ T cell apoptosis and subsequent granuloma disruption (21). (3) Depletion of Mtb-specific CD4+ T cells by HIV-1 increases the risk of latent TB reactivation (22, 23). Most of the previous studies pertaining to host derived soluble factors in HIV-1/Mtb co-infection highlighted the cytokines with limited information on chemokines. Since the chemokine biology itself is a large area and to discuss the relevance of each chemokine sub-families is beyond the scope of current review. Thus, we focus on most relevant C–C chemokines associated with each pathogen and in a setting of HIV-1/Mtb-co-infection.

## Chemokines in HIV-1 Pathogenesis and Disease Progression

HIV-1 induced inflammatory chemokines exhibit dual function. For example, C–C chemokine CCL3, CCL4, CCL5, and C–X–C chemokine CXCL12 (SDF-1α) function to block the entry of R5 and R4 HIV-1 strains (24–26) thereby preventing HIV-1 replication. While another C–C family member, CCL2 has been suggested to support HIV-1 replication (3, 17, 18). The most compelling evidence in support of chemokines in HIV-1 pathogenesis is exhibited by the individuals homozygous for 32 bp-CCR5 deletion conferring resistance toward HIV-1 (27) and recently reported stem cell transplant study from individual with 32 bp-CCR5 deletion to HIV-1 infected patient showing a long-term HIV-1 control (28). Thus early production of above suppressive chemokines in the lymph nodes can benefit host by restricting HIV-1 dissemination (29). This unique feature of HIV-1 suppressive chemokines made the basis of developing CCR5 antagonist Maraviroc, which has now progressed to clinical practice (30).

CCL2, is a strong chemo-attractant of CCR2+ monocytes/macrophages and CD4+ T cells (9, 10). In human peripheral blood, CCL2 is mainly produced by circulating monocytes, in particular by the CD14+ CD16+ inflammatory monocyte subsets of HIV-1 patients (3, 31). Clinical data including ours showed an elevated CCL2 levels in the serum and cerebrospinal fluid (CSF) and that significantly correlates with plasma viral load of HIV-1 patients (3, 18, 32–34). Further we showed a selective up-regulation CCL2 mRNA and serum CCL2 by HIV-1 viremic than aviremic patients (3), suggesting differential CCL2 response by host depends on the status of HIV-1 replication. This observation was further strengthened in a case study where a highly viremic HIV-1+ patient who received a short-term prednisolone treatment for severe uveitis, displayed a drastic viral load reduction paralleled with declined CCL2 expression (35). Another evidence supporting the association of viremia with CCL2 production is recently reported in the CSF specimens of “Elite controllers” (EC) (36). ECs are a rare group of HIV-1+ individuals with persistently suppressed viral load (37). Strikingly, both EC and HIV-negative individuals show lower level of serum CCL2 compared to HIV-1+ individuals. Based on our own and others findings a hypothetical model on impact of CCL2 in HIV-1 pathogenesis is described elsewhere (4, 17). This explains (1) recruitment of HIV-1 permissive monocytes/macrophages and CD4+ T cells at the site of infection for new rounds of replication (feed-back loop model), (2) induction of HIV-1 co-receptor CXCR4 by CCL2 (38), (3) CCL2-mediated polarization of helper T cell (Th0) toward Th2 phenotype (39), (4) IL-4 induction CXCR4 expression on resting CD4 T cells (40), and (5) enhanced HIV-1 progeny release by CCL2 (34). Thus, a selective inhibition of CCL2 could provide an attractive anti-inflammatory intervention in HIV/AIDS.

## Chemokines in *M. tuberculosis* Infection

It is well established now that Mtb infection can occur throughout the course of HIV-1 infection (2) and that eventually results in diagnostic and preventive challenges. The protective immunity against Mtb is mainly driven by CD4+ T cells and macrophages, supported by a network of inflammatory cytokines and chemokines. Among these IFN-γ and TNF-α are the two major cytokines conferring protective immunity against Mtb (41). TNF-α, in addition to macrophage activation also induces secretion of several C–C and C–X–C chemokines including CCL2 (42, 43). Availability of animal models including mice, guinea pig, and non-human primate (NHP) have immensely contributed to our understanding of inflammatory reactions in Mtb infection (21). For example in murine model of Mtb infection, secretion of C–X–C chemokines, CXCL3, and CXCL5 (44) lead to influx of CXCR2+ neutrophils and NK cells while CXCL13 (45) recruits follicular helper (Tfh) CXCR5+ T cell into lung to provide immune protection against TB (46). Similarly, chemokine CCL5 tends to play an important role in T cell priming by recruiting lymphocytes into the lung, thereby helping in controlling murine Mtb infection. Recent study on BAL samples of HIV-1/Mtb co-infected patients showed a significant correlation of viremia with CCL5 and its receptor CCR5 (47) suggesting persistent HIV-1 replication in the lung drives activation of local T cells as evident by high expression of CCR5 in HIV/latent Mtb co-infection.

Owing to its strong chemotactic and pro-inflammatory properties, CCL2 has been shown to participate in granuloma formation (48) and to certain extent protection against Mtb (49). While other Mtb-induced C–C chemokine CCL3, CCL4, and CCL5 has been described to inhibit bacilli growth (50). Among the C–X–C chemokine, CXCL8 (IL-8), is reported to be the most important soluble factor which got elevated and recruits monocytes and lymphocytes in the lung of TB patients (51). Looking closely on CCL2 role in Mtb infection, studies have described induction of CCL2 by both the BCG and Mtb in *in vitro* as well as in BCG vaccinated individuals *in vivo* (52). Interestingly, a high CCL2 level was found to be associated with disease severity of TB patients (52). In addition to pathogen and induced soluble factors, host genetic make-up is considered as key factor in determining the Mtb infection susceptibility and progression to active TB. Recent population genetics studies have described the association of CCL2 polymorphism and TB disease. For example individuals with CCL2-2518G allele show significant association with risk of developing active TB in Asian and Hispanic population (53).

## Potential Impact of Mtb-Induced CCL2 in HIV-1 Pathogenesis

Previous studies have described the exploitation of lung and pleura-specific cellular environment by HIV-1 for replication and subsequent induction of inflammatory mediators such as CCL2 (19). In pulmonary TB infection, bacilli enter the lung via respiratory pathway which are subsequently encountered and phagocytosed by alveolar macrophages and DC (20). During this event activated macrophages secrete TNF-α to control Mtb bacilli growth (54). It is should be noted that TNF-α is also known to activate HIV-1 replication (55), that means the detrimental effects of one pathogen proving beneficial to the other. Nevertheless, Mtb has been shown to induce HIV-1 replication in acutely and chronically infected macrophages or T cells (56) as well as in alveolar macrophages and lymphocytes of HIV-1 infected individuals (57, 58). These effects are clinically displayed as high viral load in the plasma of HIV-1/Mtb co-infected (59, 60) as well as in BAL (61) of TB patients, suggesting Mtb could support HIV-1 replication by manipulating the lung microenvironment.

**Figure 1:**
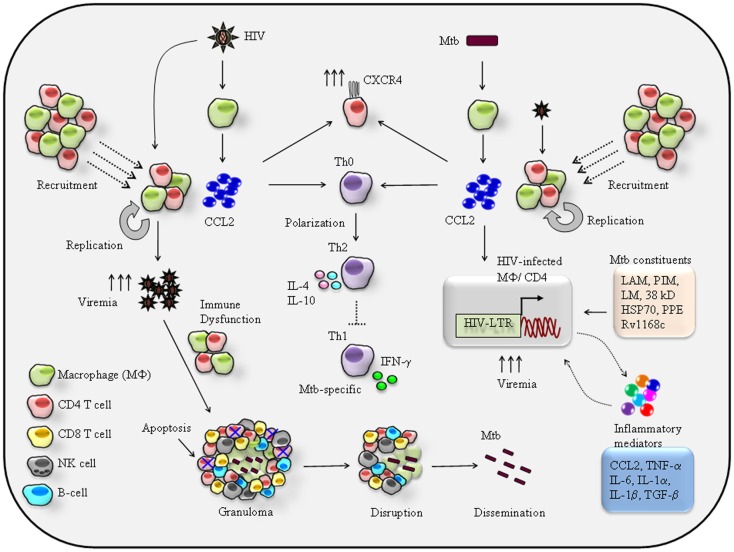
**Proposed mechanisms of reciprocal effects of CCL2-mediated immuno-pathogenesis of HIV/Mtb co-infection**. HIV-1 infection of alveolar macrophage releases CCL2 that recruits monocytes/macrophages and CD4+ T cells at the site of infection hence increase the pool of HIV-1 permissive cells for new round of replication-the feed-back loop model (4, 17) eventually persistence of a high viremia in the BAL. CCL2 can acts on resting CD4+ T cells to induce expression of HIV-1 co-receptor CXCR4, thereby rendering them susceptible to infection by X4 strains (38). CCL2 known to trigger differentiation of Th0 toward Th2 phenotype (39) via CCL2–CCR2 axis. Therefore, in the lung a high CCL2 creates a Th2 dominant environment that presumably suppresses Mtb-specific Th1 response. Persistence HIV-1 replication and high viremia in the lung impairs the macrophage and CD4+ T cell effector function against Mtb. Most importantly, the targeted apoptosis of CD4+ T cells leads to granuloma disruption leading to reactivation and dissemination of latent TB (21). On other hand secretion of CCL2 by Mtb infection may shares the similar effects like cellular recruitment, CXCR4 induction, and suppression of Mtb-specific Th1 immune response very much similar to those imposed by HIV-1. In addition to CCL2, Mtb and its cell wall constituents like lipo-arabinomannan (LAM), phosphatidylinositol (PIM), lipomannan (LM), and 19-kD Mtb protein (56), the 38-kD glycoprotein and HSP70 recognition by TLR4 (66) proline–proline-glutamic acid (PPE) protein Rv1168c (67, 68) by pattern recognition receptor (PPR) and C-type lectin receptor (69) result in secretion of pro-inflammatory cytokines and chemokines including TNF-α (5), CCL2 (54, 70, 71), IL-1α/β (5, 72), IFN-γ (56, 73), and IL-6 (74–76) that can trigger HIV-1 replication by activating HIV-LTR of the infected macrophages or CD4+ T cells eventually a high viremia. While the secreted inflammatory molecules can act in autocrine manner to activate HIV-LTR. Thus, productions of these inflammatory mediators lead to local immune reaction that eventually enhances the severity of HIV/Mtb comorbidity.

A hypothetical model based on available data can be is summarized (Figure 1). Firstly recruitment of HIV-1 permissive CCR2+ monocytes/macrophages and CD4+ T cells (20) by CCL2 released from Mtb-infected alveolar macrophages (55, 62) increases the risk of HIV-1 infection. Secondly, CCL2-mediated activation of HIV-LTR (long-terminal repeats) as shown in infected macrophages and CD4+ T cells (63) also results in induction of pro-inflammatory genes such as TNF-α, CCL2, and IL-6 (59, 60), that mean CCL2–CCR2 axis has dual effects on HIV-1 infected cells by inducing HIV-LTR and pro-inflammatory genes. Studies have also shown, activation of latent HIV-1 by Mtb-purified protein derivatives (PPD) in the alveolar macrophages of infected patients (64). Thus, one can argue that both CCL2 and bacilli can affect the HIV-1 replication in HIV-1/Mtb co-infection scenario (Figure 1). A number of studies have described Mtb-derived products to trans-activate HIV-LTR coupled with pro-inflammatory genes expression upon recognition by cell surface molecules (Figure 1). This event is regulated by several innate molecular signaling pathways (6) including MAPKinases, NFkB, C/EBPs, and very recently identified NFAT5 (65). A sustained and prolonged activation of signaling pathways and subsequent secretion of inflammatory mediators including CCL2 may cause chronic inflammation and that may prove fatal to HIV-1/Mtb co-infected individuals.

## HIV-Induced CCL2 in Latent TB Reactivation and Pathogenesis

As estimated nearly one third population of world is living with Mtb infection and individuals with HIV/AIDS are at high risk of developing active TB (77). Entry of Mtb bacilli into the lung, trigger early immune response where DC captures Mtb and migrate to nearby draining lymph nodes to start Mtb-specific adaptive immune responses (78). This leads to activation, expansion, and functional maturation of Mtb-specific CD4+ T cells that home to the site of primary infection in lung and activates innate immune cells such as macrophages to release IFN-γ and TNF-α to control infection (55, 79). Some of the potential mechanisms by which HIV-1 facilitate Mtb pathogenesis are, up-regulation of Mtb receptor on macrophages (80, 81), impaired leukocyte recruitment (82), altered Th1/Th2 balance (83), and impaired TNF-α mediated macrophage apoptosis (84). In fact, the greater impact of HIV-1 in Mtb disease is the reactivation of latent TB by disruption of granuloma, a feature commonly associated with immune compromised conditions such as HIV/AIDS (85). Based on *in vitro* and *ex vivo* clinical findings some of the potential pathways by which HIV-1 induced CCL2 can contribute to TB reactivation and associated pathology (Figure 1) include (1) HIV-1 infected alveolar macrophages secreted CCL2 recruits CCR2+ leukocytes including macrophages, CD4+ T cells, and NK cells to participate in immune reactions. This gives HIV-1 opportunity to infect and replicate within these freshly recruited permissive cells resulting in high viremia as detected in the BAL fluid of HIV-1/Mtb co-infected patients (7, 21, 86, 87). (2) Persistence of high HIV-1 viremia causes macrophage dysfunction to kill Mtb (20). (3) Entry of HIV-1 into granuloma causes CD4+ T cell apoptosis, depletion, and disorganization of granuloma (85, 88, 89), resulting in Mtb dissemination associated with extra-pulmonary TB manifestations. (4) IFN-γ producing CD4+ T cells are crucial for Mtb control (21) thus, depletion of Mtb-specific CD4+ T cells by HIV-1 certainly is an important factor to increase the risk of latent TB reactivation (80, 81). (5) Given that CCL2 favors Th2 response which is evident from CCL2^−/−^ mice that confer resistance against parasitic infection (22, 23) under this scenario, a higher CCL2 level in the BAL of HIV-1/Mtb patients (39) will generate a Th2 dominant environment that presumably suppresses Mtb-specific IFN-γ mediated Th1 immunity (Figure 1). Taken together, this hypothetical model explains the CCL2 mediated on-going lung-specific HIV-1 and Mtb interplay and a mechanistic insight how HIV-1 and Mtb reciprocate each other in a setting of HIV-1/Mtb co-infection.

## IRIS Associated Complications in HIV-1/Mtb Co-Infection

Although highly active anti-retroviral therapy (HAART) dramatically declines the HIV/AIDS-associated morbidity and mortality by restoring CD4 T cell counts in HIV-1 infected individuals but an unwanted phenomena of immune reconstitution inflammatory syndrome (IRIS) hampers the successful treatment of HIV-1/Mtb co-infection (90, 91). There are two forms of TB-IRIS, the “paradoxical” which occurs in patient receiving anti-TB drugs before HAART and “unmasking” TB-IRIS in patients with initiation of HAART without prior clinical symptoms of TB (92). Despite serious clinical efforts, the pathogenic mechanism of IRIS is largely unknown. Some of the most widely accepted potential mechanisms includes (a) the magnitude of immune restoration, (b) the antigenic burden, and (c) the host genetic susceptibility. Moreover, early prediction of developing either forms of IRIS may benefit susceptible individuals by introducing preventive approaches. In this regard, some of the predictive chemokine markers such as CCL2, CXCL8, and CXCL10 have been suggested (93). Of which, the pro-inflammatory CCL2 is argued as a strong predictor of TB-IRIS in HIV patients after commencing HAART. Further studies are required to understand the mechanism and identification of a set of biomarkers to predict IRIS for improved disease management.

## Conclusion and Perspectives

As a major public health issue it is critical to understand and exploit the beneficial and detrimental effects imposed by each pathogen on host survival in a situation of HIV-1/Mtb co-infection. Under this complex scenario, inflammatory mediators tend to play pivotal role in containing pathogens and disease progression. Although Mtb-specific CD4+ T cells are critical for controlling active TB at the same time they are prone to attack by HIV-1 (20). Therefore, in addition to reconstitution of CD4+ T cells by anti-retroviral and anti-TB therapy regimens, strategies should be developed to reduce CCL2 expression to contain severity of co-infection. Due to ethical limitations, a direct study on HIV-1/Mtb co-infected individuals is not feasible, animal models could prove an alternative and valuable tool for such studies. Efforts have been made in this regard to generate CD4+ T cell-deficient mouse model mimicking HIV/AIDS-associated features (81) and humanized mice (94) to study HIV-1 pathogenesis and behavior within granuloma (95). Moreover, several NHP models of HIV-1/Mtb co-infection have also been developed (96) including new cynomolgus macaque model to address SIV induced reactivation of latent TB (86, 97, 98). Further studies focusing CCL2 in these animal models will allow us to unravel the mechanism of CCL2-mediated co-infection pathogenesis and consequences of HIV-1: Mtb interactions on disease outcome. We hope this will lead to manipulate CCL2 as an anti-inflammatory intervention point in HIV/AIDS-related comorbidities.

## Conflict of Interest Statement

The authors declare that the research was conducted in the absence of any commercial or financial relationships that could be construed as a potential conflict of interest.
